# Location and extent of disease predicts outcome of neurofibromatosis type 1-related pediatric low-grade gliomas

**DOI:** 10.1093/noajnl/vdaf050

**Published:** 2025-02-28

**Authors:** Sneha M Chaturvedi, Arohi Saxena, Ahmad Hassan, Ali Mian, Chelsea Kotch, Nicole M Brossier

**Affiliations:** Department of Genetics, Washington University School of Medicine, St. Louis, Missouri, USA; Department of Pediatrics, Washington University School of Medicine, St. Louis, Missouri, USA; Department of Pediatrics, Washington University School of Medicine, St. Louis, Missouri, USA; Department of Radiology, Washington University School of Medicine, St. Louis, Missouri, USA; Division of Oncology, Children’s Hospital of Philadelphia, Philadelphia, Pennsylvania, USA; Department of Pediatrics, Washington University School of Medicine, St. Louis, Missouri, USA

**Children with neurofibromatosis type 1 (NF1) are at increased risk of developing low-grade gliomas (LGGs) in multiple brain locations.^1^ In children with sporadic pilocytic astrocytoma, location impacts the rate of progression and accumulation of neurological deficits,^2^ with supratentorial midline (SM, including optic pathway) tumors exhibiting worse neurologic morbidity over time. We performed a multi-institutional retrospective cohort study to determine how location affects progression and functional morbidity in NF1-LGG. We observed an increased risk of symptomatic relapse and more neurologic deficits over time in optic pathway tumors compared to other locations; this risk was most pronounced in children with deep extensive gliomas (DEGs).**

Children with NF1 are at risk for the development of LGG, which arises most commonly in the optic pathway (optic pathway glioma: OPG) and brainstem (BS).^3,4^ When symptomatic, OPG typically manifests with visual deficits or endocrinopathies, while LGG in other locations may present with headache, seizure, weakness, or other neurologic impairments.^1,3–5^ One-third to one-half of NF1-LGG require treatment, including surgical resection and/or chemotherapy.^6^ Studies in sporadic gliomas have found that tumor location is associated with outcomes such as tumor progression and functional deficit burden.^2,7^ However, it is unclear whether tumor location is similarly associated with outcomes in NF1 patients, who often have a more indolent clinical course than individuals with sporadic LGG. For these reasons, we conducted a multi-center retrospective cohort study to evaluate the impact of tumor location on long-term outcomes for children with NF1-LGG.

**Figure 1.**
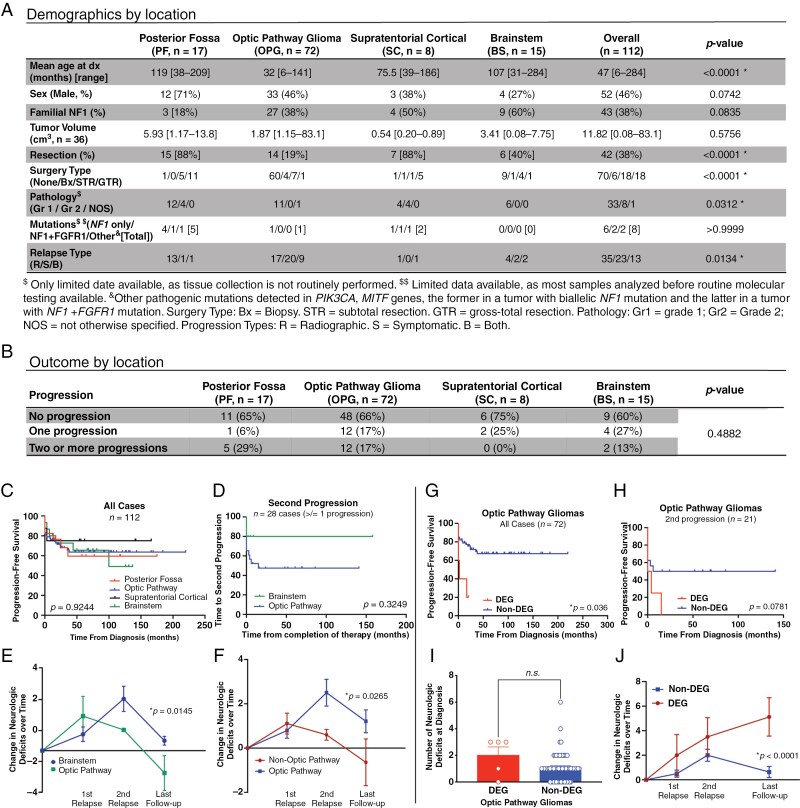
NF1 optic pathway gliomas exhibit greater rates of symptomatic progression and increased burden of neurologic deficits over time. Multi-institutional retrospective chart review included 99 patients with NF1-related CNS tumors (*n* = 112 tumors) who received neuro-oncologic treatment (surgery, chemotherapy, or radiation) between 1999 and 2021 at St. Louis Children’s Hospital and Children’s Hospital of Philadelphia. Per standard clinical practice, tumors were predominantly classified as low-grade gliomas radiographically and resection/biopsy was only performed in a minority of cases. Neurological deficits were quantified at initial treatment, progression and last follow-up. One point was awarded for vision loss, each endocrinopathy, dysphagia, dysarthria, cranial nerve palsies, unilateral upper extremity (UE) weakness, unilateral lower extremity (LE) weakness, unilateral facial weakness, seizures, ataxia, and nystagmus. At each progression, one point was added for each worsening neurological symptom or new symptom and one point was removed for each resolved symptom. 0.5 points were subtracted if a symptom was improving but not resolved. (A) While there were no sex differences in glioma incidence, OPG presented at an earlier age compared to PF tumors (*p* < 0.0001). Tumor volumetric analysis, performed in a subset of patients from St. Louis Children’s Hospital, revealed no significant difference by location. Resection (either gross total or subtotal) was most common in PF and SC groups (*p* < 0.0001), likely due to better surgical accessibility. Histology was only available in a subset of patients; all had biopsy-confirmed low-grade glioma, with more grade 2 tumors occurring in the PF and SC regions (*p* = 0.0312). Given higher rates of surgery in these areas, this may represent sampling bias. Tumor molecular analysis was only available in a subset of patients; most tumors had biallelic *NF1* loss, with a smaller subset showing *NF1* mutation in combination with *FGFR1* mutation. There was insufficient data to draw inferences based on location. Progression type was classified as radiological (R), symptomatic (S), or both (B); OPGs displayed higher rates of symptomatic progression than non-OPGs (*p* = 0.0134). Statistical significance was determined by 1-way ANOVA with Tukey post hoc test for multiple comparisons. (B) Incidence of progression did not differ significantly between groups. (C) PFS did not differ significantly amongst all 4 tumor locations. (D) In tumors that progressed one or more times, OPGs (*n* = 23) showed a non-significant trend toward worse PFS at second progression than BSGs (*n* = 5; Log-rank Mantel–Cox test, *p* = 0.3249). (E) OPGs were associated with acquisition of more neurologic deficits over time than BSGs (2-way ANOVA, interaction *p* = 0.0145). (F) In tumors that progressed one or more times, OPGs acquired more neurologic deficits over time than non-OPGs (*2*-way ANOVA, interaction *p* = 0.0265). These included worsening vision, development of new endocrinopathies, extremity weakness, gait changes, and seizures, among others. (G) Non-DEG OPGs had a significantly longer PFS compared to DEGs (Log-rank Mantel–Cox test, *p* = 0.036). (H) DEGs demonstrated a trend toward shorter time to second progression (Log-rank Mantel–Cox test, *p* = 0.078). (I) DEGs and non-DEGs exhibited no difference in the number of neurological deficits at diagnosis (*t*-test, *p* = 0.0721). (J) In tumors that progressed one or more times, DEGs had a significantly increased neurological deficit burden over time (mixed effects analysis, interaction *p* = 0.0008) compared to non-DEG. This effect was pronounced at last follow-up.

This multi-institutional retrospective chart review received approval from the institutional review boards at St. Louis Children’s Hospital and Children’s Hospital of Philadelphia. The study population included 99 children 18 years of age or younger at the time of initial diagnosis of NF1-LGG who received treatment for their target tumor between 1999 and 2021. Per consensus recommendations for NF1-associated glioma,^8^ tumors were classified as probable LGG by radiographic features; biopsies were not standardly performed. Patients with multiple gliomas in distinct locations had each tumor counted separately, with 112 total NF1-LGG included in this study. Each NF1-LGG was categorized into one of 4 locations: posterior fossa (PF, *n* = 17, 15%; not including brainstem), optic pathway (OPG, *n* = 72, 64%, including the optic nerves, chiasm, radiations, and hypothalamus), supratentorial cortical (SC, *n* = 8, 7%), and brainstem (BSG, *n* = 15, 13%) (**Figure 1A**). OPGs were further classified into DEGs or non-DEGs, with DEGs defined as tumors involving the optic pathway and bilateral temporal lobes, thalami and basal ganglia.^1^ Tumor progression was determined based on radiographic or symptomatic criteria. Neurologic deficits (described in the figure legend) were determined at baseline, each progression, and at the last follow-up.

In the analytic cohort, OPGs presented at a younger age compared to PF tumors (32 vs. 119 months; **Figure 1A**). No significant association was identified between tumor location and biological sex or familial NF1 status. PF tumors were significantly more likely to undergo surgical intervention than OPGs (88% vs. 19%). OPGs displayed higher rates of symptomatic relapses, such as visual acuity decline, when compared against all non-OPG tumors (63% vs. 39%).

There was no association between tumor location and the number of progressions over time (**Figure 1B**) or the time to first progression (**Figure 1C**). These data contrast with sporadic tumors, where SM and BS pilocytic astrocytoma have shorter progression-free survival (PFS) due to higher rates of subtotal resection.^2^ Additional analyses comparing OPG versus BSG subgroups were performed, given higher rates of treatment of NF1-LGG in these locations.^1,3,4^ Mean longitudinal follow-up was similar between the 2 (77 vs. 79 months, respectively). OPGs trended toward a shorter time to second progression when compared to the BSG subgroup, but this did not reach statistical significance, likely due to insufficient sample size (**Figure 1D**). OPGs showed more neurologic deficits over time than BSGs (**Figure 1E**) and all non-OPGs (interaction, *p* = 0.0071; not shown). In patients that had undergone at least one relapse, OPGs also showed a trend toward more severe neurologic deficits over time than BSGs (not shown) and a statistically significant increase in neurologic deficits over time when compared to all non-OPGs (**Figure 1F**). From the entire patient cohort, one patient with BSG died before the end of the study period, consistent with previously described overall survival rates for patients with NF1-LGG.^9^

NF1-related DEGs have been associated with poorer PFS.^1^ We evaluated the OPG group by the extent of disease, dividing patients into those that met the criteria for DEG (*n* = 5) versus those that did not (*n* = 67). All DEGs were infiltrative across multiple adjacent brain regions at first treatment, although not all met the criteria for DEG at that time. All DEG tumors were identified in males, a sex bias not seen previously. DEGs demonstrated a significantly shorter PFS (**Figure 1G**), which aligns with previous studies.^1^ Four of the 5 patients with DEG demonstrated at least 2 progression events, with a median time to second progression of 4.75 months (range 0–15). There was a trend toward shorter PFS at the second progression than non-DEG (**Figure 1H**; *p* = 0.0781). DEGs did not have a greater number of neurologic deficits at diagnosis (**Figure 1I**) but were associated with increased accumulation of neurologic burden over time (**Figure 1J**).

This analysis had several limitations, including sample size, abstraction of progression and neurologic deficits by retrospective chart review, the inclusion of both symptomatic and asymptomatic (radiographic) progressions, and comparatively few progressive tumors identified outside the optic pathway. Despite these limitations, our study identified a higher rate of symptomatic disease progression and poorer longitudinal outcomes in individuals with NF1-OPG compared to NF1-LGG in other locations, confirming the SM location as a risk factor for poorer disease outcomes in NF1-LGG. Within the OPG cohort, DEGs appear to be at particularly increased risk for recurrent progression and increased number of neurologic deficits over time, confirming the extent of tumor infiltration as a clinical feature associated with tumor progression^10^ and suggesting that these patients in particular may benefit from alternative treatment strategies. Prospective studies, such as the ongoing NF1-LGG and NF1-OPG natural history studies, will be needed to confirm tumor location and extent of disease as prognostic factors in NF1-LGG.
